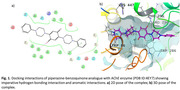# Novel piperazine‐benzoquinone derivative as a possible lead molecule selectively targeting AChE for the management of dementia in Alzheimer’s disease

**DOI:** 10.1002/alz.087881

**Published:** 2025-01-09

**Authors:** Pragati Silakari, Poonam Piplani, Amit Kumar, Amarjot Kaur Grewal

**Affiliations:** ^1^ Chitkara College of Pharmacy, Chitkara University, Rajpura, Punjab India; ^2^ Panjab University, Chandigarh, Chandigarh India

## Abstract

**Background:**

The present study recapitulates the potency of the novel synthesized piperazine‐benzoquinone derivative as a lead molecule selectively targeting AChE along with the antioxidative potential for the management of cognitive decline in Alzheimer’s disease.

**Method:**

Novel piperazine‐benzoquinone derivative was synthesized implementing appropriate synthetic procedures and was characterized by various spectral and elemental techniques. The purity of this synthetic analogue was ascertained by TLC, melting point determination and elemental analyses. Further, the compound was evaluated for its selective *in vitro* acetylcholinesterase (AChE) and butyrylcholinesterase (BChE) inhibitory potential at different concentrations using mice brain homogenate as the source of the enzyme. The analogue further evaluated for their antioxidant potential using DPPH radical scavenging and hydrogen peroxide radical scavenging protocols at five different concentrations and further studied against behavioural alterations using step down passive avoidance and escape learning protocol at a dose of 0.5 mg/kg with reference to the standard drug donepezil. Scopolamine at a dose level of 2.0 mg/kg was used as an inducer of dementia in the animal model. The *ex vivo* acetylcholinesterase (AChE) inhibition using mice brain homogenate as the source of the enzyme were also performed. Biochemical estimation of the markers of oxidative stress (lipid peroxidation, superoxide dismutase, glutathione and catalase) has also been carried out to determine the role of the synthesized molecule on the scopolamine induced oxidative damage.

**Result:**

A synergistic effect of two structural components *viz*. the benzoquinone moiety and the cyclic secondary amino subunits on the inhibitory activity against AChE has been anticipated. The compound exhibited selective acetylcholinesterase activity (**IC_50_ 06.795 µM**), appreciable selectivity index value (**0.353**) and significant antioxidant potential. The *ex vivo* AChE inhibition assays were also in conformity with the behavioural studies data along with the docking results showing important interactions with AChE protein (**Figure 1**)

**Conclusion:**

Outcomes depicted the anti‐amnesic effect of the synthesized conjugate on scopolamine‐induced memory impairment probably related to mediation of cholinergic system and their effective antioxidant activity. Results obtained were found to be appreciable and provided promising lead which can be further explored structurally and pharmacologically, presenting a favourable scenario for the development of selective AChE inhibitors.